# Molecular epidemiology and phylogenetic analysis of Hepatitis B virus in a group of migrants in Italy

**DOI:** 10.1186/s12879-015-0994-9

**Published:** 2015-07-25

**Authors:** Umbertina Villano, Alessandra Lo Presti, Michele Equestre, Eleonora Cella, Giulio Pisani, Marta Giovanetti, Roberto Bruni, Elena Tritarelli, Massimo Amicosante, Alba Grifoni, Carmelo Scarcella, Issa El-Hamad, Maria Chiara Pezzoli, Angeletti Silvia, Anna Rita Ciccaglione, Massimo Ciccozzi

**Affiliations:** Viral Hepatitis Unit, Department of Infectious, Parasitic and Immune-Mediated Diseases, Istituto Superiore di Sanità, Rome, Italy; Epidemiology Unit, Department of Infectious, Parasitic and Immune-Mediated Diseases, Istituto Superiore di Sanità, Rome, Italy; Department of Cell Biology and Neurosciences, Istituto Superiore di Sanità, Rome, Italy; Center for Immunobiologicals Research and Evaluation, Istituto Superiore di Sanità, Rome, Italy; Department of Biomedicine and Prevention, University of Rome “Tor Vergata”, Rome, Italy; Department of Biology, University of Rome “Tor Vergata”, Rome, Italy; Department of Infectious Diseases, Spedali Civili General Hospital, Brescia, Italy; Brescia Local Health Authority, Brescia, Italy; Clinical Pathology and Microbiology Laboratory, University hospital Campus Biomedico, Rome, Italy

**Keywords:** HBV, Phylogeny, Phylodynamics, Molecular epidemiology, Migrants

## Abstract

**Background:**

Hepatitis B virus infection (HBV) is widespread and it is considered a major health problem worldwide. The global distribution of HBV varies significantly between countries and between regions of the world. Among the many factors contributing to the changing epidemiology of viral hepatitis, the movement of people within and between countries is a potentially important one. In Italy, the number of migrant individuals has been increasing during the past 25 years. HBV genotype D has been found throughout the world, although its highest prevalence is in the Mediterranean area, the Middle East and southern Asia. We describe the molecular epidemiology of HBV in a chronically infected population of migrants (living in Italy), by using the phylogenetic analysis.

**Methods:**

HBV-DNA was amplified and sequenced from 43 HBV chronically infected patients.

Phylogenetic and evolutionary analysis were performed using both maximum Likelihood and Bayesian methods.

**Results and conclusion:**

Of the 43 HBV S gene isolates from migrants, 25 (58.1 %) were classified as D genotype.

Maximum Likelihood analysis showed an intermixing between Moldavian and foreigners sequences mostly respect to Italian ones. Italian sequences clustered mostly together in a main clade separately from all others. The estimation of the time of the tree’s root gave a mean value of 17 years ago, suggesting the origin of the tree back to 1992 year. The skyline plot showed that the number of infections softly increased until the early 2005s, after which reached a plateau. Comparing phylogenetic data to the migrants date of arrival in Italy, it should be possible that migrants arrived in Italy yet infected from their country of origin. In conclusion, this is the first paper where phylogenetic analysis and genetic evolution has been used to characterize HBV sub genotypes D1 circulation in a selected and homogenous group of migrants coming from a restricted area of Balkans and to approximately define the period of infection besides the migration date.

**Electronic supplementary material:**

The online version of this article (doi:10.1186/s12879-015-0994-9) contains supplementary material, which is available to authorized users.

## Background

Hepatitis B virus (HBV) is a circular, partially double-stranded DNA virus of about 3.7 k bases, of the family *Hepadnaviridae;* its genome has four overlapped open reading frames (ORFs) that codify for: envelope (S/Pre-S), core (C/pre-C), polymerase (P) and X (HBV-X) proteins [1, 2]. Infection with HBV affects the liver and results in a broad spectrum of disease outcomes: the infection can spontaneously resolve and lead to protective immunity, result in a chronic infection and cause acute liver failure [3]. HBV infection is widespread and it is considered a major health problem worldwide with approximately one third of the world’s population that has been exposed to the virus, and an estimated 350 million people are chronically infected [4, 5].

Every year there are over 4 million acute clinical cases of HBV, and about 25 % of, 1 million people a year, die from chronic active hepatitis, or primary liver cancer [World Health Organization. http://www.who.int/csr/disease/hepatitis/whocdscsrlyo20022/en/index8.html#51].

In Europe the HBV prevalence rates are variables between different countries: in general, countries in the south-eastern part are still at high level of endemicity, while western countries report low prevalence of HBV infection [3].

Despite the recent decrease in the rate of new cases, about 7–8,000 new diagnoses are made every year in Europe [3].

The global distribution of HBV varies significantly between countries and between regions of the world. Among the many factors contributing to the changing epidemiology of viral hepatitis, the movement of people within and between countries is a potentially important one [6]. Migration has historically played a role in influencing demographic changes and public health.

More than 70 % of the estimated 25 million foreigners living in the European Union’s countries come from Eastern and South-Eastern Europe and North Africa. However, migrants to the European Union (EU) are diverse in terms of their country of origin, and the number of immigrants from Latin America, Asia and Sub-Saharan Africa is growing (http://www.ecdc.europa.eu).

In Italy, the number of migrant individuals has been increasing during the past 25 years. It has been estimated that, by the end of 2011, 5 million foreign individuals were living in Italy. Of these, 27.4 % were from European (EU) countries of the EU Community, 23,4 % from EU countries not belonging to the EU Community, 22.1 % from Africa, 18,8 % from Asia and 8.3 % from America [7].

Ten genotypes (A-J) that differs genetically by at least 8 % have so far been identified for HBV [8], some of which further segregate into sub-genotypes with a mean genetic distance of about 4 % [9]. The genotypes and sub-genotypes have a distinct ethno-geographical distribution. Some are ubiquitous, such as genotype A, which is present in north-western Europe, North America Central Africa and Asia [10], and genotype D, which has been found throughout the world, although its highest prevalence is in the Mediterranean area, the Middle East and southern Asia, particularly India [10, 11].

Other genotypes are locally restricted to more limited geographical areas [8]. The two genotypes responsible for the majority of infections in Europe are genotype A (mainly subgenotype A2) in the north-western part of Europe and genotype D (mainly subgenotypes D1, D2 and D3) in the south eastern Europe and the Mediterranean area [9].

The aim of the present study was to describe the molecular epidemiology of HBV in a chronically infected population of migrants living in Italy, by using the phylogenetic analysis.

The temporal dynamics was performed by using a Bayesian approach.

## Methods

### Patients

Serum samples were from 43 HBV chronically infected patients followed at the Service of International Medicine of Brescia’s Local Health Authority, in a period from 2004 to 2010. The Service of International Medicine (SIM) was created initially in 1990 to provide a free of charge basic medical assistance for undocumented migrants.

Patients included in the study were HBsAg positive with either HBeAg reactivity or HBV-DNA values greater than 2,000 IU/ml. Upon these criteria they were defined as having active chronic hepatitis B.

The study was approved by Ethical Committee of Brescia Health Local Authority and a written informed consent was obtained from all participating subjects [12]. All migrants from Moldavia arrived in Italy from 2003 to 2008.

### HBV DNA extraction, amplification and sequencing

HBV-DNA was extracted from serum sample at the National Institute of Health (Istituto Superiore di Sanita) and genotyping was performed on HBV-DNA positive sera. Viral DNA was extracted from 200 μl of serum using the EZ1 Virus Mini Kit v.2.0 (Qiagen Hilden, Germany) following the manufacturer’s instructions.

HBV-DNA was amplified by real-time polymerase chain reaction (PCR) with the Platinum Taq DNA Polymerase (Invitrogen by Life Technologies Corporation). Single set of primers corresponding to HBV S gene was used as follow:5′-AGGCGGGGTTTTTCTTGTTGACAA-3′(sense; nt 201–224 nt) and 5′-TTAGGRTTYAAATGTATACCCA-3′(antisense; nt 842–821). The fragment amplified by PCR was 600 base pairs (bp). The PCR conditions were: initial denaturation at 94 °C for 1 min and 30 s, followed by 30 cycles of denaturation at 94 °C for 30 s, annealing at 52° for 30 s, extension at 72 °C for 1 min. A final elongation step of 5 min at 72 °C was included at the end of the amplification cycles. Both negative and positive controls were included in each PCR run to ensure the absence of contamination and monitor amplification efficiency. The PCR products were analyzed on 1,2 % agarose gel stained with ethidium bromide.

The PCR products were purified using the QIAquick PCR Purification Kit (Qiagen Hilden, Germany) in accordance with the manufacturer’s instructions. Sequencing reactions were performed using the GenomeLab DTCS Quick Start KiT (Beckman Coulter, Inc., Fullerton, CA) and were run on an automated DNA sequencer (Beckman Coulter, Inc., Fullerton, CA).

A total of 43 sequences were successfully obtained. The sequences were submitted to GenBank (Accession Numbers from KR871232 to KR871274).

### Sequence dataset

Four different dataset were built. The first one contained 43 HBV S gene sequences from migrants plus 105 genotype specific reference sequences downloaded from the National Centre for Biotechnology Information (NCBI) (http://www.ncbi.nlm.nih.gov/.); this dataset has been used to establish the genotype.

The second dataset included 17 Moldovan HBV sequences previously classified as D1 genotype plus 228 HBV D1 genotype sequences downloaded from NCBI and it was used to to estimate the S gene mean evolutionary rate.

The reference sequences were selected according to the following inclusion criteria: (i) sequences had already been published in peer-reviewed journals; (ii) the subtype assignment of each sequence was certain; (iii) the state of origin and the sampling date were known and clearly established in the original publication.

The third dataset included only the 17 HBV D1 genotype Moldovan sequences and was used to perform the population dynamics and to perform the time scale tree.

The fourth dataset was composed of 418 HBV D1 genotype sequences, 17 of which were the 17 Moldovan isolates and 401 were D1 genotype foreign sequences (68 sampled from Italy, 15 from Bulgaria, 35 from Lebanon, 108 from Iran, 2 from Uzbequistan, 3 from Tajikistan, 3 from Belarus, 47 from Turkey, 4 from Poland, 5 from China, 3 from Egypt, 17 from Sudan, 14 from India, 1 from Latvia, 8 from Mongolia, 2 from Pakistan, 49 from Russia, 11 from Tunisia, 1 from France, 4 from Belgium and 1 from Germany) downloaded from NCBI and selected according to the above inclusion criteria.

### Likelihood mapping

The phylogenetic signal generated by each dataset was investigated by means of the likelihood mapping analysis of 10,000 random quartets generated using TreePuzzle as already described [13]. A likelihood map consists of an equilateral triangle containing dots representing the likelihoods of the three possible un-rooted trees for a set of four sequences (quartets) randomly selected from the dataset: the dots close to the corners or at the sides represent, respectively, tree-like (fully resolved phylogenies in which one tree is clearly better than the others) or network-like phylogenetic signals (three regions in which it is not possible to decide between two topologies). The central area of the map represents a star-like signal, (the region in which the star tree is optimal). When using this strategy, if more than 33 % of the dots fall into the center of the triangle, the data are considered unreliable for the purposes of phylogenetic inference.

### Phylogenetic analysis

The sequences of all dataset were aligned using Clustal X and manually edited by Bioedit as already described [14]. The evolutionary model was chosen for each dataset as the best-fitting nucleotide substitution model in accordance with the results of the hierarchical likelihood ratio test (HLRT) implemented in Modeltest software version 3.7, as already described [14]. The isolate genotype in the first data set was determinated by using maximum likelihood (ML) and HKY + I + G as evolutionary model by Phyml v 3.0 [15].

The evolutionary rate was estimated on the second data set by HKY + I + G evolutionary model by using BEAST software 1.7.4 [14].

The evolutionary model for the third data set was HKY + I + G [14].

The forth dataset to investigate the eventual intermixing between foreign and Moldavian sequences used GTR + I + G as the best evolutionary model in ML approach [16].

The statistical robustness and reliability of the branching order within the phylogenetic trees was confirmed by the bootstrap analysis, considering as significant statistical support a bootstrap value > 70 % and posterior probability (pp) in Bayesian analysis (pp > 90 %).

### Evolutionary rate estimate and time –scaled phylogeny reconstruction

To estimate the evolutionary rate on the second data set both a strict and relaxed clock with an uncorrelated log normal rate distribution were compared. A Bayesian Markov chain Monte Carlo (MCMC) method implemented in BEAST package 1.7.4 [17, 18] was used. As coalescent priors, three parametric demographic models of population growth (constant size, exponential, and expansion growth) and a Bayesian skyline plot (BSP, a non-parametric piecewise- constant model) were compared.

The MCMC chains were run for at least 50 million generations, and sampled every 5,000 steps. Convergence was assessed on the basis of the effective sampling size (ESS) after a 10 % burn-in [18]. Only ESS values of > 250 were accepted. Uncertainty in the estimates was indicated by 95 % highest posterior density (95 % HPD) intervals, and the best fitting models were selected using a Bayes factor (BF, using marginal likelihoods) implemented in BEAST [18]. In accordance with Kass and Raftery [19], the strength of the evidence against H0 (null hypothesis) was evaluated as follows: 2 ln BF < 0 no evidence; 2–6 weak evidence; 6–10 strong evidence; and > 10 very strong evidence. A negative 2 ln BF indicates evidence in favor of H0. Only values ≥ 6 were considered significant.

The time-scaled phylogenetic tree on the third dataset was reconstructed by using the Bayesian Markov Chain Monte Carlo approach implementing the evolutionary model selected by ModelTest, setting the evolutionary rate to the value previously estimated and the best fitting models (so as for the clock and the demographic model) were selected using a Bayes factor (BF, using marginal likelihoods).

### Demographic history

The demographic history was analyzed on the third dataset on an individual basis by comparing the four coalescent models (constant, exponential, expansional, and BSP) and implementing a relaxed molecular clock model under the conditions described above.

## Results

### Likelihood mapping analysis

The phylogenetic noise of each data set was investigated by means of likelihood mapping. The percentage of dots falling in the central area of the triangles was 10.6 %, 25.9 %, 27 % and 29 % respectively for the first, second, third and fourth dataset: as none of the dataset showed more than 30 % of noise, all of them contained a sufficient phylogenetic signal (Additional file 1: Figure S1, Panel a, b, c, d).

### Phylogenetic analysis

Maximum Likelihood phylogenetic analysis of the first dataset (Additional file 2: Figure S2) identified different statistically supported cluster (bootstrap > 70 %).

The 43 HBV S gene sequences obtained from migrants were classified as follow: 25 (58.1 %) genotype D, 1 (2.3 %) genotype E, 7 (16.3 %) genotype A, 3 (7 %) genotype C, 4 (9.3 %) genotype B and 3 (7 %) sequences were unclassified.

Specifically, of the 7 HBV genotype A isolates, 4 (57.1 %) isolates were classified as subgenotype A2 and 3 (42.9 %) isolates were classified as subgenotype A3. Amog the 25 genotype D sequences, 6 (24 %) were classified as subgenotype D2, 1 isolate (4 %) as subgenotype D3, 18 (72 %) as subgenotype D1 (17 of the 18 D1 subgenotype isolates, were from Moldova).

Maximum Likelihood analysis on the the forth data set showed an intermixing between Moldavian and foreigners sequences (Additional file 3: Figure S3).

Most of the Italian sequences clustered together in a main clade separately from all others.

### Evolutionary rate estimate and Bayesian analysis

**Fig. 1:**
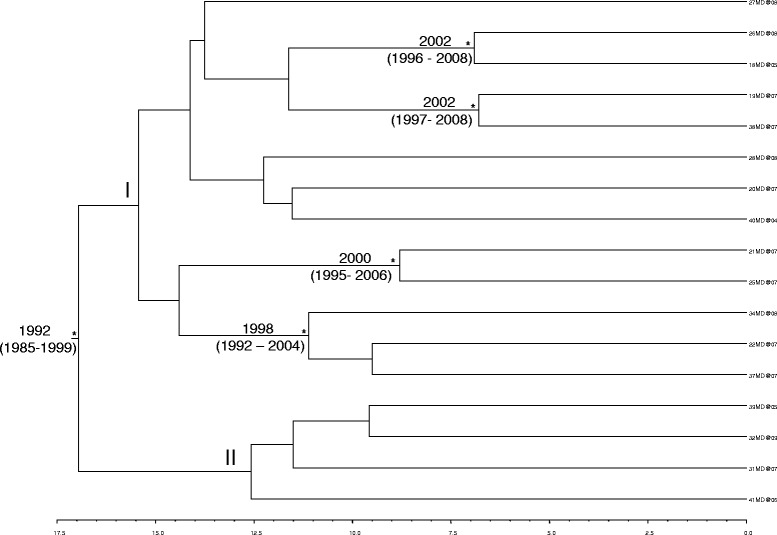
Bayesian phylogenetic tree of 17 Moldavian HBV S gene sequences. The time of the most recent common ancestor, with the credibility interval based on 95 % highest posterior density interval (95 % HPD), was reported in years. Scale years is reported at the bottom of the figure

Implementing strict and relaxed molecular clocks, MCMC searches were performed, on the second dataset, and the marginal likelihoods of the obtained trees were compared using a BF to select the best model and parameter values. BF analysis showed that the relaxed clock fitted the data significantly better than the strict clock (2lnBF between the strict and relaxed clock was 716.556 in favour of the second). Under the relaxed clock, the BF analysis showed that the Bayesian skyline plot (BSP) was better than other models (2lnBF > 120). The estimated mean value of the HBV S gene evolutionary rate for the second dataset was 4.4 × 10^−4^ (95 % HPD: 2.6 × 10^−4^ - 6.2 × 10^−4^). Figure 1 showed the reconstruction of the time scaled Bayesian tree of the 17 D1 Moldavian sequences (third data set).

Comparing data from the Bayesian tree (Fig. 1) with the date of arrival in Italy, we observed that whereas only 3/17 (17.7 %) D1 Moldavian sequences were from migrants arrived on the year 2003 most of the sequences 14/17 (82.3 %) were from migrants arrived in Italy from 2004 to 2008. In particular, 6/17 (35.2 %) migrants arrived on 2004; 3/17 (17.7 %) on 2005; 4/17 (23.5 %) on 2006 and 1/17 (5.9 %) on 2008. The estimation of the time of the tree’s root gave a mean value of 17 years ago (95 % HPD between 24 and 10 years ago) this suggests that the origin of the tree goes back 1992 year (C.I. 1985–1999), (Fig. 1).

### Demographic history

**Fig. 2:**
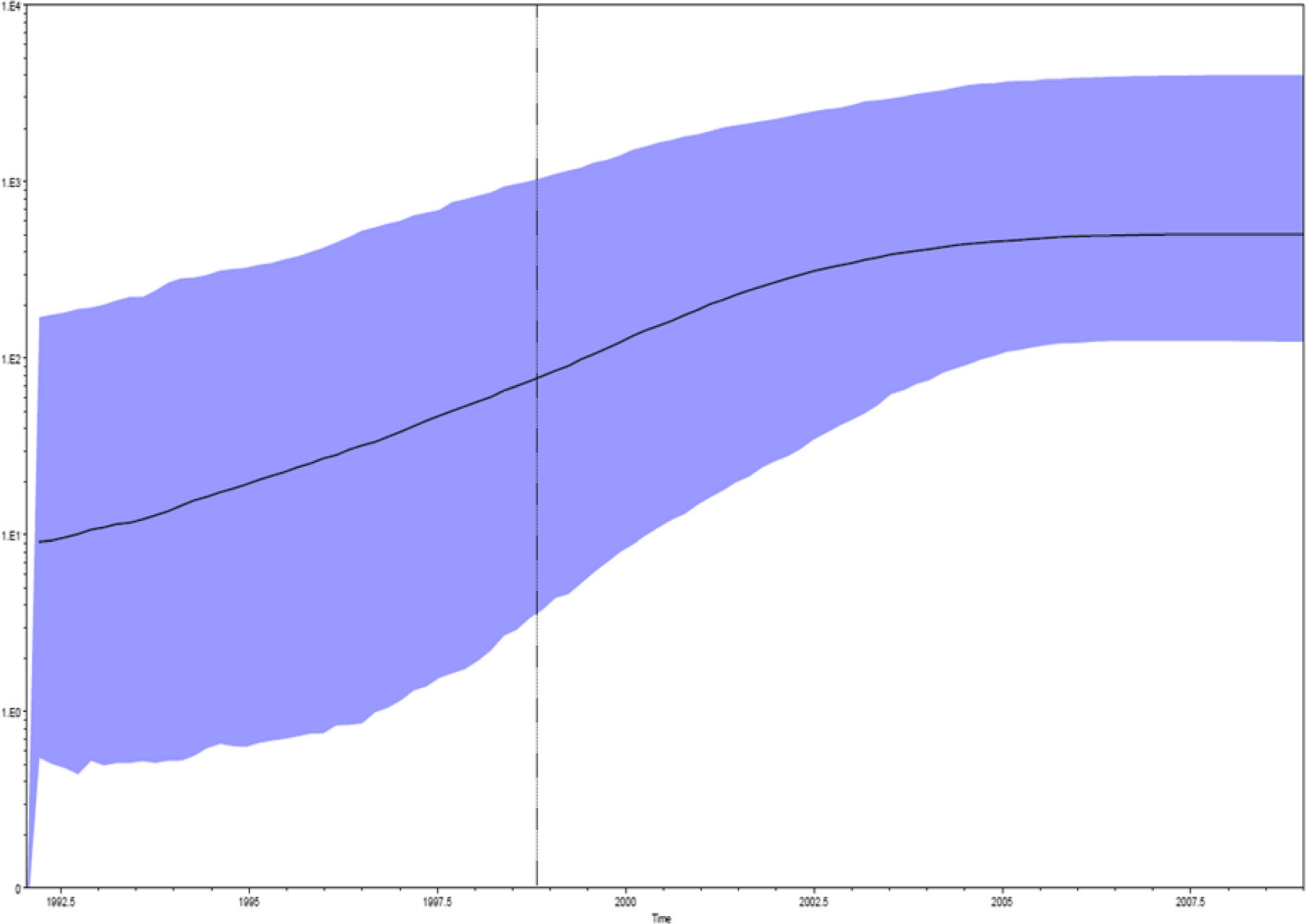
Bayesian skyline plot (BSP) of the 17 HIV Moldavian HBV S gene sequences. The effective number of infections is reported on the Y-axis. Time is reported on the X-axis. The colored area corresponds to the credibility interval based on 95 % highest posterior density interval (HPD)

The evolutionary demography of the Moldovan HBV D1 sequences (third dataset) was also investigated by population dynamics analysis. BF analysis showed that the BSP was favourite with respect to other models (2 ln BF >11.6). The skyline plot (Fig. 2) showed that the number of infections softly increased until the early 2005s, after which reached a plateau.

## Discussion

HBV virus is widespread in the world. The different route of transmission and the efficient dissemination permitted its spreading in the world. Long-term persistence, long incubation period and low frequency of symptoms helped HBV to maintain the high incidence rate in many countries. In the present study, the genetic diversity and demographic history of HBV in 43 HBV chronically infected migrants, resident in north of Italy, by using a Bayesian coalescent-based framework, was investigated. The most prevalent genotype in north-eastern Europe, the Mediterranean basin, northern Africa, and the Middle East is genotype D. It is highly prevalent in the Indian sub-continent and a group of island in the Indian Ocean with high endemic levels of HBV [20]; and it has also been identified in Oceania [10].

Most migrants living in Italy come from areas with intermediate or high prevalence of HBV infection such as Eastern Europe (23.4 % of the total number of documented migrants in Italy), Africa (22.1 %) and Asia (18.8 %) and the first four ranking Countries are Morocco, China, India and Albania [12].

Palumbo *et al.* showed that 9.3 % - 10.7 % of individuals recently migrated to Italy and hosted in temporary camps in southern Italy tested HBsAg positive [21]. In a recent study, HBsAg reactivity and associated risk factors among migrants who accessed the Service of International Medicine of Brescia’s Local Health Authority was assessed and the prevalent genotype was D [12].

The phylogenetic analysis of the 43 HBV S gene sequences obtained from migrants showed that the most frequent genotype was sub-genotype D1 and that all isolates with sub-genotypes D1, except one, were Moldovan sequences. For this reason our analysis was focalized on the 17 D1 Moldovan sequences. D1 is the most prevalent sub-genotype in East Europe, Balkans and north Africa [22–24].

The time-scaled tree of the 17 D1 Moldavian sequences allowed to estimate the time to the most recent common ancestor (TMRCA). The root of the tree was dated back to the year 1992, thus suggesting that the HBV D1 strains circulating in Moldovan migrants originated since that date. From the 1992 ancestor the 17 D1 Moldavian sequences clustered mainly together in a branch dated recently on average from 1992 to 2008 (clade I) and consequently strains in this clade should be circulating in this frame time.

Furthermore, the evolutionary demography of the Moldovan HBV D1 sub genotypes sequences was analyzed and an exponential growth of the number of infections was observed between the early 2003 and 2005 whereas a plateau was reached in 2007 (Fig. 2). Maximum Likelihood analysis and skyline plot showed an intermixing mostly between Moldavian and foreigners as well as an exponential increasing number of infections during 2003–2005.

The analysis of the 17 D1 sequences in the fourth dataset, including the more prevalent D1 sequences from different countries, [23, 24] showed that the Moldavian sequences were intermixed and mostly correlated with Russian and other east European and Asiatic countries. Interestingly, the major part of the Italian sequences formed a clear separated clade than all other sequences.

Comparing these results to the date of arrival in Italy, it should be possible to assume that infection of migrants occurred in their country of origin. This hypothesis was also supported by the skyline plot showing that the number of infections softly increased until the early 2005s.

## Conclusions

In conclusion, this is the first paper where phylogenetic analysis and genetic evolution are used to characterize HBV sub-genotypes D1 circulation in a selected and homogenous group of migrants coming from a restricted area of East-Europe and to approximately define the period of infection.

### Availability of supporting data

All the supporting data are included as additional files.

## Additional files

Additional file 1: Figure S1. Likelihood mapping of the first (panel a), second (panel b), third (panel c) and fourth (panel d) HBV S gene dataset. The dots inside the triangles represent the likelihood of the possible unrooted topologies for each quartet. Numbers indicate the percentage of dots in the centre of the triangle corresponding to phylogenetic noise (star-like trees). Additional file 2: Figure S2. Maximum likelihood phylogenetic analysis including 43 HBV S gene isolates sequences plus 105 genotype specific reference sequences. The tree was rooted by the midpoint rooting. Branch lengths were estimated with the best fitting nucleotide substitution model according to a hierarchical likelihood ratio test, and were drawn to scale with the bar at the bottom indicating 0.0060 nucleotide substitutions per site. One asterisk (*) along the branches represents significant statistical support for the clade subtending that branch (bootstrap value >70 %). Additional file 3: Figure S3. Maximum likelihood phylogenetic tree of the fourth dataset. The tree was rooted by the midpoint rooting. Branch lengths were estimated with the best fitting nucleotide substitution model according to a hierarchical likelihood ratio test, and were drawn to scale with the bar at the bottom indicating 0.01 nucleotide substitutions per site. One asterisk (*) along the branches represents significant statistical support for the clade subtending that branch (bootstrap value > 70 %). The most prevalent sequences are in color. Moldovan sequences were highlighted in red, Italian sequences in blue, Russian in green, Iranian in violet, Turkey in light blue and Lebanon in light green.

## Abbreviations

HBV: Hepatitis B virus
ORFs: Open reading frames
EU: European Union
SIM: Service of International Medicine
NCBI: National Centre for Biotechnology Information
HLRT: Hierarchical likelihood ratio test
ML: Maximum likelihood
pp: Posterior probability
MCMC: Markov chain Monte Carlo
ESS: Effective sampling size
HPD: Highest posterior density
BF: Bayes factor
TMRCA: Time to the most recent common ancestor

## References

[1] Hollinger FB, Liang TJ, Knipe DM, Howley PM, Griffin DE, Lamb RA, Martin MA, Roizman B (2001). Hepatitis B virus. Fields Virology.

[2] Locarnini S (2004). Molecular virology of hepatitis B virus. Semin Liver Dis.

[3] European Centre of Disease, Prevention and control ECDC. Technical report. Hepatitis B and C in the EU neighbourhood: prevalence, burden of disease and screening policies. 2010.

[4] World Health Organization (2004). Hepatitis B vaccines. Wkly Epidemiol Rec.

[5] Lavanchy D (2004). Hepatitis B, virus epidemiology, disease burden, treatment, and current and emerging prevention and control measures. J Viral Hepat.

[6] Carballo M, Cody R, O’Reilly E. Migration, Hepatitis B and Hepatitis C. international Centre for Migration Health and Development.

[7] Dossier statistico immigrazione 22nd Report. Caritas e Migranti. 2012.

[8] Zehender G, Ebranati E, Gabanelli E, Sorrentino C, Lo Presti A, Tanzi E (2014). Enigmatic origin of hepatitis B virus: an ancient travelling companion or a recent encounter?. World J Gastroenterol.

[9] Schaefer S (2007). Hepatitis B, virus taxonomy and Hepatitis B virus genotypes. World J Gastroenterol.

[10] Norder H, Courauce AM, Coursaget P, Echevarria JM, Lee SD, Mushahauar IK (2004). Genetic diversity of Hepatitis B virus strains derived worldwide: genotypes, subgenotypes and HBsAg subtypes. Intervirol.

[11] Amini-Bavil-Olyaee S, Sarrami-Forooshani R, Mahboudi F, Sabahi F, Adeli A, Noorinayer B (2007). Genotype characterization and phylogenetic analysis of hepatitis B virus isolates from irananian patients. J Med Virol.

[12] El-Hamad I, Pezzoli MC, Chiari E, Scarcella C, Vassallo F, Puoti M (2014). Point-of-care screening, prevalence, and risk factors for hepatitis B infection among 3,728 mainly undocumented migrants from non-EU Countries in Northern Italy. J Travel Med.

[13] Strimmer K, von Haeseler A (1997). Likelihood-mapping: a simple method to visualize phylogenetic content of a sequence alignment. PNAS.

[14] Ciccozzi M, Lo Presti A, Cella E, Giovanetti M, Lai A, El-Sawaf G (2014). Phylogeny of dengue and Chikungunya viruses in Al Hudayda governorate, Yemen. Infect Genet Evol.

[15] Guindon S, Dufayard JF, Lefort V, Anisimova M, Hordijk W, Gascuel O (2010). New algorithms and methods to estimate maximum-likelihood phylogenies: assessing the performance of PhyML 3.0. Syst Biol.

[16] Price MN, Dehal PS, Arkin A (2010). FastTree 2-approximately maximum likelihood trees for large alignments. Plos ONE.

[17] Drummond AJ, Rambaut A, Shapiro B, Pybus OG (2005). Bayesian coalescent inference of past population dynamics from molecular sequences. Mol Biol Evol.

[18] Drummond AJ, Rambaut A (2007). BEAST: Bayesian evolutionary analysis by sampling trees. BMC Evol Biol.

[19] Kass RE, Raftery AE (1995). Bayes factors. J Am Stat Assoc.

[20] Murhekar MV, Murhekar KM, Sehgal SC (2008). Epidemiology of hepatitis B virus infection among the tribes of Andaman and Nicobar Islands, India. Trans R Soc Trop Med Hyg.

[21] Palumbo E, Scotto G, Faleo G (2007). Prevalence of HBV – genotype in immigrants affected by HBV- related chronic active hepatitis. Arc Gastroenterol.

[22] Ozaras R. Sexually transmitted diseases. Hepatitis B. ESCMID.

[23] Yousif M, Kramvis A (2013). Genotype D of Hepatitis B virus and its subgenotypes: an update. Hepathology Res.

[24] Ozaras R, Inanc Balkan I, Yemisen M, Tabak F (2015). Epidemiology of HBV subgenotypes D. Clin Res Hepathol Gastroenterol.

