# Erratum to: Molecular epidemiology and phylogenetic analysis of Hepatitis B virus in a group of migrants in Italy

**DOI:** 10.1186/s12879-015-1245-9

**Published:** 2015-11-17

**Authors:** Umbertina Villano, Alessandra Lo Presti, Michele Equestre, Eleonora Cella, Giulio Pisani, Marta Giovanetti, Roberto Bruni, Elena Tritarelli, Massimo Amicosante, Alba Grifoni, Carmelo Scarcella, Issa El-Hamad, Maria Chiara Pezzoli, Silvia Angeletti, Anna Rita Ciccaglione, Massimo Ciccozzi

**Affiliations:** Viral Hepatitis Unit, Department of Infectious, Parasitic and Immune-Mediated Diseases, Istituto Superiore di Sanità, Rome, Italy; Epidemiology Unit, Department of Infectious, Parasitic and Immune-Mediated Diseases, Istituto Superiore di Sanità, Rome, Italy; Department of Cell Biology and Neurosciences, Istituto Superiore di Sanità, Rome, Italy; Center for Immunobiologicals Research and Evaluation, Istituto Superiore di Sanità, Rome, Italy; Department of Biomedicine and Prevention, University of Rome “Tor Vergata”, Rome, Italy; Department of Biology, University of Rome “Tor Vergata”, Rome, Italy; Department of Infectious Diseases, Spedali Civili General Hospital, Brescia, Italy; Brescia Local Health Authority, Brescia, Italy; Clinical Pathology and Microbiology Laboratory, University hospital Campus Biomedico, Rome, Italy

After publication of the original article [1] it came to the authors’ attention that Silvia Angeletti’s name was displayed incorrectly as ‘Angeletti Silvia’. The correct spelling of Silvia Angeletti’s name has been updated in the original article and is corrected with this erratum.
