# Supplementary figures and images for: Molecular epidemiology and phylogenetic analysis of Hepatitis B virus in a group of migrants in Italy
Source: BMC Infect Dis. 2015 Jul 25;15:287. doi: 10.1186/s12879-015-0994-9 (PMC4514992; doi:10.1186/s12879-015-0994-9)

a)

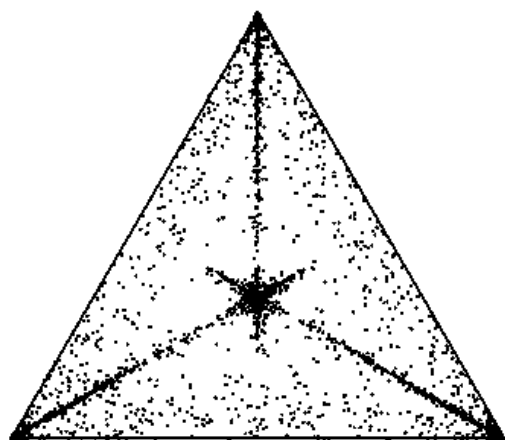

10.6 %

b)

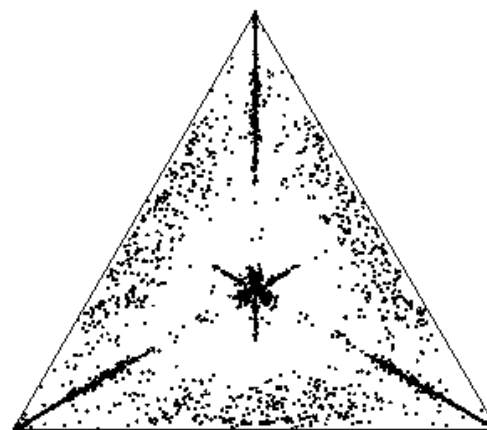

25.9 %

c)

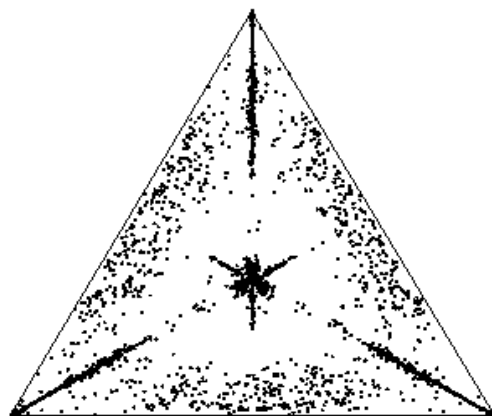

27 %

d)

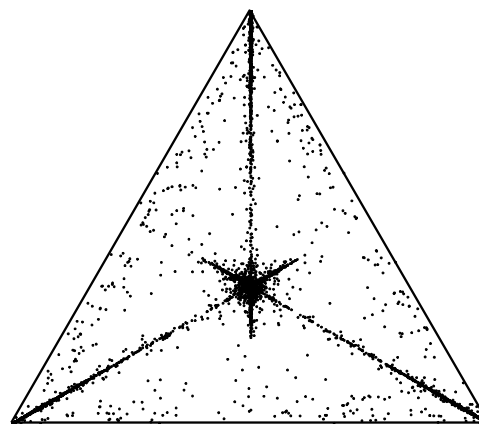

29 %

Supplement: Additional file 1: Figure S1. — Likelihood mapping of the first (panel a), second (panel b), third (panel c) and fourth (panel d) HBV S gene dataset. The dots inside the triangles represent the likelihood of the possible unrooted topologies for each quartet. Numbers indicate the percentage of dots in the centre of the triangle corresponding to phylogenetic noise (star-like trees). [file 12879_2015_994_MOESM1_ESM.pdf]

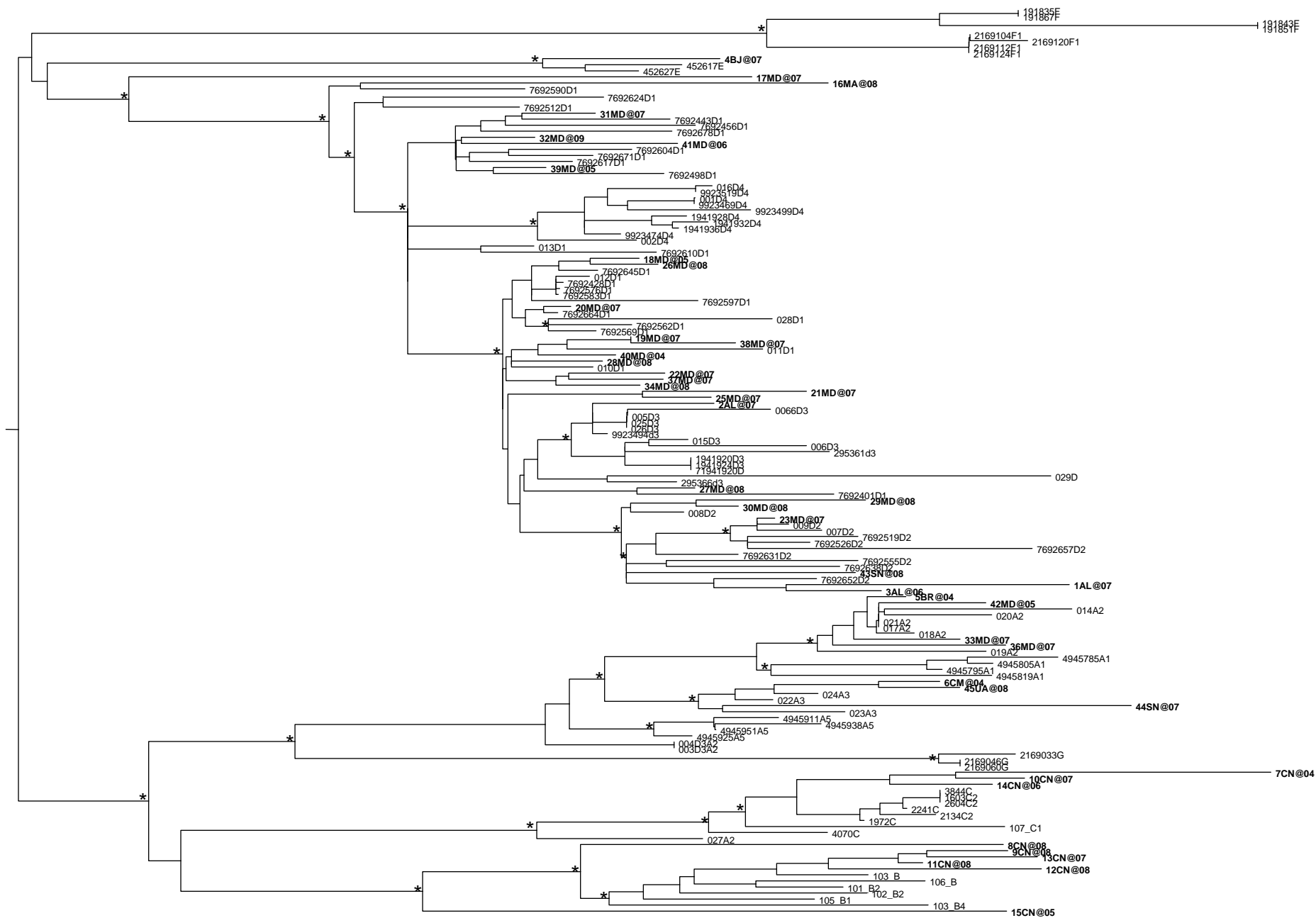

Supplement: Additional file 2: Figure S2. — Maximum likelihood phylogenetic analysis including 43 HBV S gene isolates sequences plus 105 genotype specific reference sequences. The tree was rooted by the midpoint rooting. Branch lengths were estimated with the best fitting nucleotide substitution model according to a hierarchical likelihood ratio test, and were drawn to scale with the bar at the bottom indicating 0.0060 nucleotide substitutions per site. One asterisk (*) along the branches represents significant statistical support for the clade subtending that branch (bootstrap value >70 %). [file 12879_2015_994_MOESM2_ESM.pdf]
