# Supplementary material for: Molecular epidemiology and phylogenetic analysis of Hepatitis B virus in a group of migrants in Italy
Source: BMC Infect Dis. 2015 Jul 25;15:287. doi: 10.1186/s12879-015-0994-9 (PMC4514992; doi:10.1186/s12879-015-0994-9)
Supplement: Additional file 3: Figure S3. — Maximum likelihood phylogenetic tree of the fourth dataset. The tree was rooted by the midpoint rooting. Branch lengths were estimated with the best fitting nucleotide substitution model according to a hierarchical likelihood ratio test, and were drawn to scale with the bar at the bottom indicating 0.01 nucleotide substitutions per site. One asterisk (*) along the branches represents significant statistical support for the clade subtending that branch (bootstrap value > 70 %). The most prevalent sequences are in color. Moldovan sequences were highlighted in red, Italian sequences in blue, Russian in green, Iranian in violet, Turkey in light blue and Lebanon in light green. [file 12879_2015_994_MOESM3_ESM.pdf]

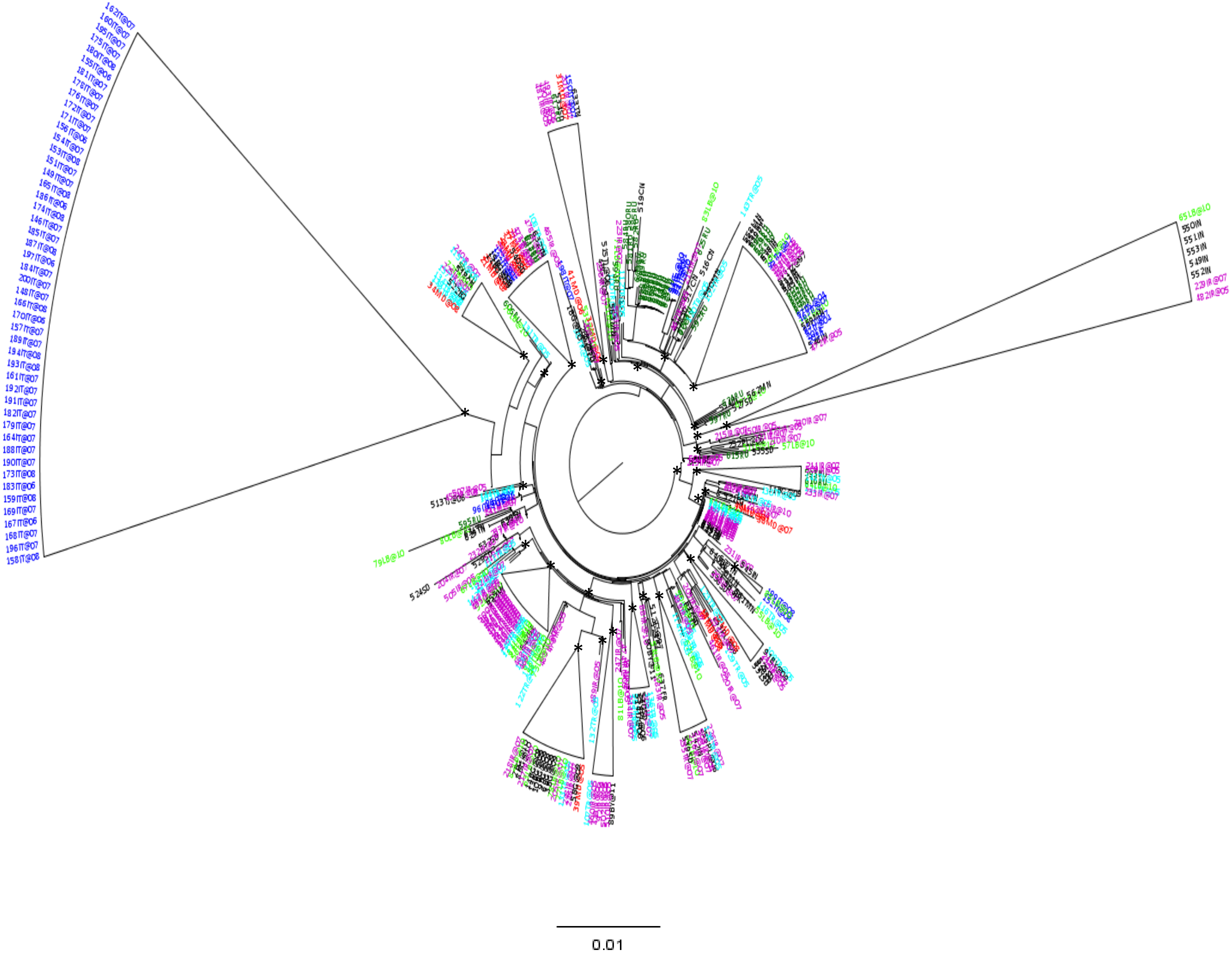
0.01
